# Ultrasonographic median nerve cross-section areas measured by 8-point "inching test" for idiopathic carpal tunnel syndrome: a correlation of nerve conduction study severity and duration of clinical symptoms

**DOI:** 10.1186/1471-2342-11-22

**Published:** 2011-12-21

**Authors:** Shu-Fang Chen, Cheng-Hsien Lu, Chi-Ren Huang, Yao-Chung Chuang, Nai-Wen Tsai, Chiung-Chih Chang, Wen-Neng Chang

**Affiliations:** 1 Department of Neurology, Kaohsiung Chang Gung Memorial Hospital and Chang Gung University College of Medicine, Kaohsiung, Taiwan; 2 Department of Biological Science, National Sun Yat-Sen University, Kaohsiung, Taiwan

## Abstract

**Background:**

Incremental palmar stimulation of the median nerve sensory conduction at the wrist, the "inching test", provides an assessment with reference to segments proximal and distal to the entrapment. This study used high-resolution ultrasonography (US) to measure the median nerve's cross-section areas (CSAs) like the "inching test" and to correlate with the nerve conduction study (NCS) severity and duration of carpal tunnel syndrome (CTS).

**Methods:**

Two hundred and twelve (212) "CTS-hands" from 135 CTS patients and 50 asymptomatic hands ("A-hands") from 25 control individuals were enrolled. The median nerve CSAs were measured at the 8-point marked as *i*4, *i*3, *i*2, *i*1, *w*, *o*1, *o*2, and *0*3 in inching test. The NCS severities were classified into six groups based on motor and sensory responses (i.e., negative, minimal, mild, moderate, severe, and extreme). Results of US studies were compared in terms of NCS severity and duration of clinical CTS symptoms.

**Results:**

There was significantly larger CSA of the NCS negative group of "CTS-hands" than of "A-hands". The cut-off values of the CSAs of the NCS negative CTS group were 12.5 mm^2^, 11.5 mm^2 ^and 10.1 mm^2 ^at the inlet, wrist crease, and outlet, respectively. Of the 212 "CTS-hands", 32 were NCS negative while 40 had minimal, 43 mild, 85 moderate, 10 severe, and two extreme NCS severities. The CSAs of "CTS-hands" positively correlated with different NCS severities and with the duration of CTS symptoms. By duration of clinical symptoms, 12 of the 212 "CTS-hands" were in the 1 month group; 82 in >1 month and ≤12 months group, and 118 in >12 months group. In "inching test", segments *i*4-*i*3 and *i*3-*i*2 were the most common "positive-site". The corresponding CSAs measured at *i*4 and *i*3, but not at *i*2, were significantly larger than those measured at points that were not "positive-site".

**Conclusions:**

Using the 8-point measurement of the median nerve CSA from inlet to outlet similar to the "inching test" has positive correlations with NCS severity and duration of CTS clinical symptoms, and can provide more information on anatomic changes. Combined NCS and US studies using the 8-point measurement may have a higher positive rate than NCS alone for diagnosing CTS.

## Background

Carpal tunnel syndrome (CTS) is a common entrapment neuropathy of the median nerve [1]. Currently, nerve conduction study (NCS) is used to confirm the diagnosis and indicate the level of the lesion [2,3]. Among various NCS methods for evaluating CTS, incremental palmar stimulation of the median nerve sensory conduction at the wrist, the so-called "inching test", permits an assessment with reference to nerve segments proximal and distal to the entrapment [4]. Aside from NCS, peripheral nerve ultrasonography (US) is a promising complementary tool [3,5-9]. However, because of different US methods, the measured values of the median nerve in CTS also vary [7-10]. This study introduced an 8-point measurement of the median nerve's cross-sectional area (CSA) from inlet to outlet similar to those performed in the "inching test". The measured CSAs were also compared to NCS severity and duration of CTS symptoms.

## Methods

This prospective case-control study conducted over a period of three years (2006-2008) enrolled 160 participants and 262 hands. Of the 160 participants, 135 with 212 hands had clinical symptoms of CTS ("CTS-hands") while the other 25 participants of 50 hands were asymptomatic ("A-hands") and acted as controls. Of the 135 symptomatic participants, 105 were women and 30 were men, aged 22-83 years (mean, 52.2 ± 11.7 years). Their body height ranged from 142 to 177 cm (mean, 158 ± 6.3 cm), body weight 40 to 86 kg (mean, 60.7 ± 8.7 kg), and body mass index 17.5 to 35.3 (mean, 24.1 ± 3.4). The basic information of the controls is listed in Table 1. The term "A-hands" was defined as a hand with normal NCS findings and not fulfilling any clinical definition of CTS.

**Table 1:** Basic information of the control participants and the patients with NCS negative "CTS" hands

	"A-hands" (n = 50)	NCS negative "CTS hands" (n = 32)	*p *value
**Sex**	14 hands in man/36 hands in woman	4 hands in man/28 hands in woman	0.100

**Age (yr)**	44.2 ± 9.8 (46, 25-68)	48.6 ± 11.9 (49, 27-73)	0.135

**BH (cm)**	163 ± 7.2 (163, 148-174)	159 ± 6.9 (157.5, 150-177)	0.004*

**BW (kg)**	61.0 ± 8.1 (60, 46-76)	59.6 ± 10.8 (56, 46-86)	0.282

**BMI**	22.8 ± 3.0 (22.7, 17.5-28.3)	23.6 ± 4.4 (22.9, 17.5-35.3)	0.711

Abbreviations: A-hands, asymptomatic hands; CTS, carpal tunnel syndrome; NCS, nerve conduction study; BH, body heigh; BW, body weight; BMI, body mass index; Mean ± standard deviation (Median, minimum-maximus);

*p < 0.01 by Mann-Whitney U test

In order to avoid other interfering factors, none of the 160 participants had diabetes mellitus, gout, rheumatoid arthritis, renal or liver disease, abnormal thyroid function, abnormal serum cortisol level, or elevated serum anti-nuclear antibody. None of the participants had a history of previous wrist surgery or fracture, or a history or clinical evidence of neurologic disorders (e.g. ulnar neuropathy, radiculopathy, polyneuropathy, myelopathy, or stroke) that might result in numbness or paresthesia. Participants with a variant of carpal tunnel, such as accessory muscles, bifid median nerve, and persistent median artery were also excluded. None of the female participants were pregnant at the time of the study. The hospital's Ethics Committee approved the study (IRB 100-1390B).

Two physicians (Drs CSF and TNW) previously trained by musculoskeletal radiologists and with more than three years of experience in patients with related disorders, especially those with clinical CTS, performed the US examinations. The clinical symptoms of each individual were recorded and the collected data were fully analyzed.

### Clinical definition of "CTS-hands"

In this study, CTS was defined according to the criteria of the American Academy of Neurology practice parameters as follows [11,12]:

1. Paresthesia, pain, swelling, weakness, or clumsiness of the hand provoked or worsened by sleep, sustained hand or arm position, or repetitive action of the hand or wrist that is mitigated by a change in posture or by shaking of the hand;

2. Sensory deficits in the median nerve innervated regions of the hand;

3. Motor deficit or hypotrophy of the median nerve innervated thenar muscles; and

4. Positive provocative clinical tests (positive Phalen's maneuver and/or Tinel's sign)

The term "CTS-hand" was defined as criterion 1 and one or more of criteria 2-4 were fulfilled. For comparative analysis, the duration of CTS symptoms was classified into three groups, i.e. ≤1 month, >1 and ≤12 months, and > 12 months.

### Neuro-physiologic assessment

**Figure 1:**
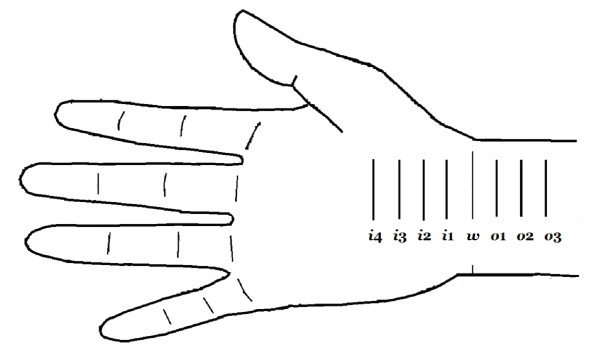
**The 8-point for recording in both "inching test" and ultrasonography**. The *i*4, *i*3, *i*2, *i*1 represent levels at 4, 3, 2, and 1 cm distal to the wrist crease in the inlet of the carpal tunnel; *w *represents the level of the wrist crease and *o*1, *o*2, and *o*3 represent levels at 1, 2, and 3 cm proximal to the wrist crease in the outlet of the carpal tunnel.

The NCS was performed for all participants according to the recommended protocol of the American Association of Electrodiagnostic Medicine (AAEM) [2] using a Nicolet Viking Select system (Nicolet Biomedical Inc. Madison, USA). All tests were done in the same room under similar temperature conditions. Skin temperature was maintained at ≥32°C. As regards NCS, the onset latency, amplitude, distance, and velocity of median, ulnar, and radial motor and sensory nerves were measured. The comparative tests included: 1) median-ulnar sensory conduction between the wrist and ring finger, 2) median sensory nerve conduction comparison between the wrist and palm, 3) median-radial sensory conduction between the wrist and thumb, and 4) antidromic sensory test using 1-cm increments of the median nerve with the wrist crease as the zero reference point extending proximally by 3 cm and distally by 4 cm. In total, eight points (Figure 1) were marked in the subsequent inching test.

The cut-off points used in the NCS were the following: 1) median nerve distal sensory latency <3.4 ms [13], 2) median nerve distal motor latency over the thenar <4.2 ms [13], 3) difference between the median and ulnar nerve distal sensory latencies <0.4 ms [14], 4) trans-carpal median motor conduction velocity <40.6 ms [15], and 5) antidromic sensory using 1-cm increments of the median nerve <0.4 ms [16]. Based on the NCS results, the CTS hands were categorized into six severity groups [17]: negative, for normal findings on all tests; minimal, for abnormal segmental or comparative tests only; mild, for abnormal digit/wrist sensory nerve conduction velocity and normal distal motor latency; moderate, for abnormal digit/wrist sensory nerve conduction velocity and abnormal distal motor latency; severe, for absence of sensory response and abnormal distal motor latency; and extreme, for the absence of motor and sensory response.

### Ultrasound assessment technique

High-resolution US was performed using a scanner with a 12/5-MHz linear array transducer for the carpal tunnel study (Philips HDI 5000; Philips Medical Systems, Bothell, WA, USA) on the same day as the NCS. During the examination, the patient sat in a comfortable position facing the examiner, with the measured forearm resting on the table, the palm supine, and fingers semi-extended in the neutral position [18]. The median nerve was first imaged in a longitudinal scan, placing the US probe at the midline between the radius and ulna with the center of the probe at the distal wrist crease, to obtain an initial general overview of the median nerve. This was then used to assist the examiner in obtaining optimal axial (cross-sectional) images. The transducer was placed directly on the patient's skin with gel.

A transverse scan, keeping the probe directly perpendicular to the long axis of the median nerve in order to ensure that the area measured indeed reflected CSA, was then performed to record the CSA (calculated by continual tracing of the nerve circumference, excluding the hyper-echoic epineurial rim) and elliptical diameters (transverse and antero-posterior). Measurements were conducted from the tunnel inlet of the forearm (*i*4, *i*3, *i*2, *i*1) to the wrist crease (*w*) and to the tunnel outlet (*o*1, *o*2, *o*3) (Figure 1).

### Statistical analysis

Data were given as mean ± standard deviation. Subsequent ANOVA analysis followed by Scheffe's multiple comparison procedures were used to calculate the mean values of CSA among different symptom duration groups, NCS types, and inching sites. To evaluate differences in CSA value at the 8-point tested between asymptomatic and CTS hands in the NCS negative group, the Mann-Whitney U test was used for comparison, as a consequence of limited data. Significance was set at *p *< 0.05 in the ANOVA and *p *< 0.01 in the Mann-Whitney U tests. The area under the ROC (Receiver Operating Characteristic) curves and the CSA cut-off-values were calculated for the negative NCS CTS hands. The Statistical Package for Social Science (SPSS Inc., version 13.0 for Windows) was used for all statistical analyses.

## Results

Based on the NCS severity classification, 32 of the 212 "CTS-hands" were in the negative group, 40 in the minimal group, 43 in the mild group, 85 in the moderate group, 10 in the severe group, and two in the extreme group. If classified according to the duration of clinical symptoms, 12 of the 212 "CTS-hands" were in the ≤1 month group, 82 in >1 month and ≤12 months group, and 118 in >12 months group.

### Comparison of CSAs at the 8-point of A-hands and NCS negative CTS-hands

The comparative results revealed significantly larger CSA of the latter group of hands at six points (*i*4, *i*3, *i*2, *i*1, *w*, and *o*3). After comparison of the CSAs under the ROC, the cut-off-values of the significant sites were 12.5 mm^2^, 11.5 mm^2^, and 10.1 mm^2^, respectively (Table 2).

**Table 2:** Comparison of CSAs measured at the 8-point of the A-hands and NCS negative CTS-hands

	CSAs		*p *value	Cut-off values of CSA	Sensitivity	Specificity
					
	A-hands (n = 50)	NCS negative CTS-hands(n = 32)				
***i*4**	11.8 ± 2.4 (11, 8-19)	14.3 ± 4.6 (13, 7-28)	0.003*	12.5	0.688	0.720
***i*3**	11.5 ± 2.3 (11, 8-19)	14.0 ± 4.3 (13, 8-28)	0.001*	12.5	0.688	0.760
***i*2**	10.9 ± 1.9 (11, 8-16)	11.9 ± 2.1 (12, 8-17)	0.033*	11.5	0.563	0.660
***I*1**	10.7 ± 1.7 (10.5, 8-15)	12.2 ± 2.7 (11.1, 7-20)	0.006*	11.1	0.500	0.680
***w***	10.7 ± 2.0 (10.5, 7-17)	12.2 ± 2.9 (12, 7-22)	0.002*	11.5	0.594	0.760
***o*1**	10.1 ± 2.2 (10, 6-16)	11.0 ± 2.3 (10, 7-16)	0.082	10.5	0.469	0.600
***o2***	10.2 ± 2.1 (10, 7-17)	10.6 ± 2.0 (10.5, 6-15)	0.164	10.5	0.500	0.600
***o*3**	9.4 ± 1.9 (9, 6-15)	10.6 ± 2.7 (11, 5-17)	0.031*	10.1	0.563	0.740

Abbreviations: CSAs, cross-section areas; A-hands, asymptomatic hands; CTS-hands, carpal tunnel syndrome hands; NCS, nerve conduction study

Markers of the 8-point: *i*4, *i*3, *i*2, *i*1, *w*, *o*1, *o*2, and *o*3 Unit of CSA = mm^2^

*p < 0.05, by Mann-Whitney U test

### Measured CSAs at the 8-point of CTS-hands with different NCS severities

**Figure 2:**
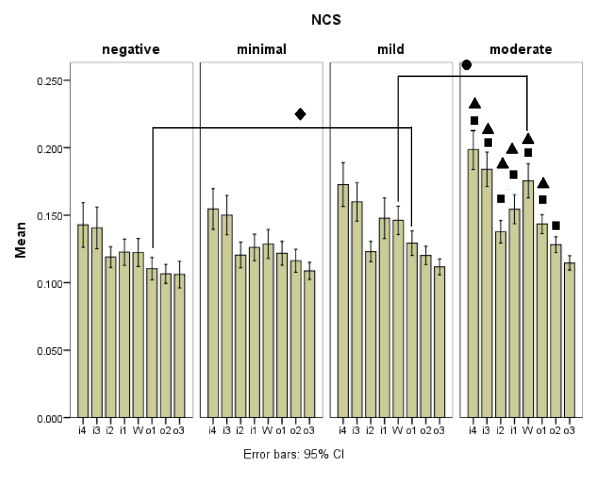
**Cross-section areas (CSAs) at the 8-point of the 212 carpal tunnel syndrome hands ("CTS-hands") with different nerve conduction study (NCS) severities**. (A) Comparison of CSAs of the NCS minimal to NCS extreme groups with the CSAs of the NCS negative group using analysis of variance (ANOVA), followed by Scheffe's multiple comparison analysis. black triangle denoting the significant difference between the NCS minimal and NCS moderate groups; black square denoting the significant difference between the NCS negative and NCS moderate groups; black circle denoting the significant difference at *w *level between the NCS mild and NCS moderate groups, black diamond denoting the significant difference at *o*1 level between the NCS negative and NCS mild groups.

**Figure 3:**
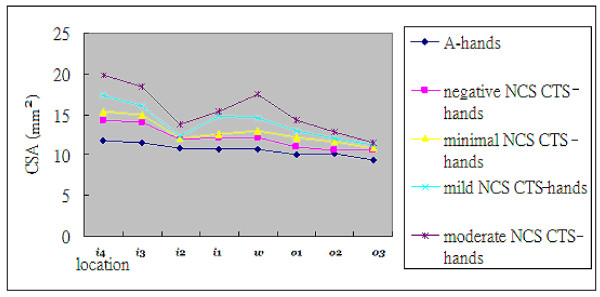
**The mean CSAs of "CTS-hands" with different NCS severities**.

The measured CSAs were compared. The NCS negative group and the mild to extreme NCS severity groups, except the minimal severity group, showed significantly larger CSA (Table 3). Because of limited case numbers, both severe and extreme groups were excluded from subsequent group comparisons; i.e. only the negative, minimal, mild, and moderate groups were included for further analysis. Mean CSAs of these four groups showed that the mean CSAs increased in accordance to severity, from negative to moderate (Figures 2 and 3).

**Table 3:** CSAs measured at the 8-point of the CTS-hands with different NCS severities (n = 212)

	Negative (n = 32)	Minimal (n = 40)	Mild (n = 43)	Moderate (n = 85)	Severe (n = 10)	Extreme (n = 2)
***i*4**	14.3 ± 4.6 (7-28)	15.4 ± 4.7 (8-30)	17.3 ± 5.3 (8-34)*	19.8 ± 6.8 (8.9-41)*	17.3 ± 8.9 (8-40.8)	15.5 ± 3.5 (13-18)
***i*3**	14.0 ± 4.3 (8-28)	15.0 ± 4.6 (8-27)	16.0 ± 4.6 (8-26)	18.4 ± 5.9 (8.9-42)*	17.3 ± 8.9 (8-40.8)	15.5 ± 3.5 (16-22)
***i*2**	11.9 ± 2.1 (8-17)	12.0 ± 3.0 (7-21)	12.3 ± 2.4 (6-18)	13.8 ± 3.8 (7-29)	13.4 ± 4.7 (8-23.6)	17.0 ± 1.4 (16-18)
***i*1**	12.2 ± 2.7 (7-20)	12.6 ± 3.1 (7-20)	14.8 ± 4.9 (8-28)	15.4 ± 5.0 (7-34.4)*	16.7 ± 6.7(11.1-34.3)*	14.5 ± 7.8 (9-20)
***w***	12.2 ± 2.9 (7-22)	12.9 ± 3.4 (7-22)	14.6 ± 3.4 (7-23) *	17.5 ± 5.8 (9-40.4)*	16.0 ± 5.7 (8.9-26)	26.5 ± 9.2 (20-33)*
***o*1**	11.0 ± 2.3 (7-16)	12.2 ± 2.8 (8-20.7)	12.9 ± 3.0 (8-20) *	14.3 ± 3.2(8.7-27.4)*	13.9 ± 4.5 (7-20.3)	22.5 ± 4.9 (19-26)*
***o*2**	10.6 ± 2.0 (6-15)	11.6 ± 2.6 (8-21)	12.0 ± 2.2 (8-17) *	12.8 ± 2.7 (7-21.3)*	13.9 ± 3.6 (9-19.9)	17.0 ± 0.0 (17-17)*
***o*3**	10.6 ± 2.7 (5-17)	10.9 ± 2.0 (7-15)	11.2 ± 1.9 (7-15)	11.5 ± 2.5 (6-19)	12.0 ± 2.2 (9-16)	19.0 ± 0.0 (19-19)*

Abbreviations: CSAs, cross-section areas; CTS-hands, carpal tunnel syndrome hands; NCS, nerve conduction study Markers of the 8-point: *i*4, *i*3, *i*2, *i*1, *w*, *o*1, *o*2, and *o*3

Unit of CSA = mm^2^

*p < 0.01, by Mann-Whitney U test (comparing CSAs of the minimal to extreme groups with the negative group)

### Frequent positive sites of the "inching test" and their correspondence to the sizes of measured CSAs

**Figure 4:**
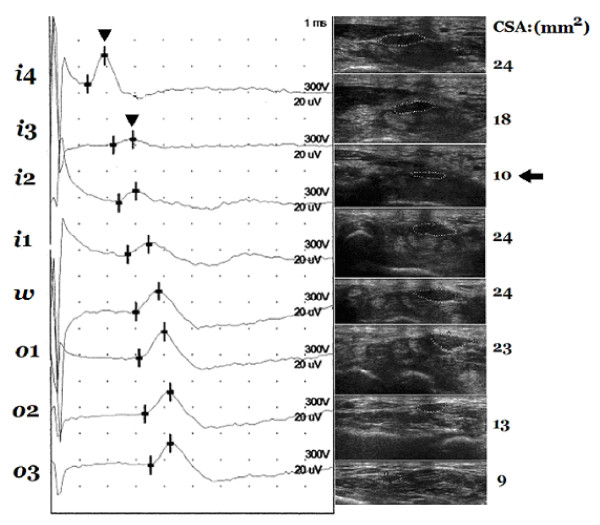
**An example of "positive-site" between *i*4 and *i*3 corresponding to the relatively smaller cross-section area (CSA) at *i*2**. The peak latencies (arrowhead) at *i*4 and *i*3 are 1.9 ms and 2.9 ms, respectively, and the difference between them is 1.0 ms, i.e. >0.4 ms. The CSA measured at *i*2 (arrow) is smaller than those measured at nearby levels. Markers of the 8-point: *i*4, *i*3, *i*2, *i*1, *w*, *o*1, *o*2, and *o*3.

The "positive-site" was defined as conduction delay (>0.4 ms) between the interval of the nearby marks in antidromic sensory test with 1 cm increments of the median nerve at the 8-point marks (Figure 4). Results showed that the most common "positive-site" were *i*4-*i*3 and *i*3-*i*2 (Table 4). The comparative results showed that CSAs corresponding to the "positive site" at *i*4-*i*3 were significantly larger than the CSAs of intervals that were not "positive-site" (Table 5). The CSAs measured at *i*2 did not show significant difference between the positive and the non-positive sites.

**Table 4:** Distributions of the positive sites in inching test of all tested hands

Inching	None	*i*4-*i*3	*i*3-*i*2	*i*2-*i*1	*i*1-*W*	*W*-*o*1	*o*1-*o*2	*o*2-*o*3	double	Total
N (%)	119	37 (25.9)	55 (38.5)	6 (4.2)	10 (7.0)	8 (5.6)	2 (1.4)	0 (0)	25 (17.4)	262

Markers of the 8-point: *i*4, *i*3, *i*2, *i*1, *W*, *o*1, *o*2, and *o*3 "double" means more than two sites existed

**Table 5:** Comparison of CSAs at the 8-points among the two most frequent positive sites and the negative site in the inching test

	none (n = 119)	i4-i3 (n = 37)	i3-i2 (n = 55)
***i*4**	13.9 ± 4.8(7-40.8)	19.5 ± 6.9 (9-37)*	19.0 ± 6.9 (8-41)*
***i*3**	13.6 ± 4.8 (8-40.8)	17.4 ± 5.0 (8-32) *	17.9 ± 6.3 (8-42)*
***i*2**	12.0 ± 3.1 (8-29)	12.4 ± 3.1 (6-19)	13.2 ± 3.4 (8-25)
***i*1**	12.4 ± 4.1 (7-34.4)	13.6 ± 4.5 (7-31)	14.6 ± 4.3 (7.4-25)*
***w***	12.5 ± 4.0 (7-31)	14.5 ± 4.6 (7-27)	15.6 ± 4.9 (7-33)*
***o*1**	11.3 ± 2.8 (6-20.7)	13.1 ± 2.9 (9-20)	13.6 ± 3.6 (8-26)*
***o*2**	11.0 ± 2.4 (6-18.5)	12.2 ± 2.4 (8-18)	12.8 ± 2.7 (8-21.3)*
***o*3**	10.5 ± 2.6 (5-19)	10.5 ± 2.3 (6-16)	11.8 ± 2.4 (8-19)*

Note: CSA, cross-section area; none, no conductive delay >0.4 ms in median inching test in centimeter across the carpal tunnel; *i*4-*i*3, conductive delay >0.4 ms in inching test between 4 cm and 3 cm distal to the wrist crease; *i*3-*i*2, conductive delay >0.4 ms in inching test between 3 cm and 2 cm distal to the wrist crease

Markers of the 8-point: *i*4, *i*3, *i*2, *i*1, *w*, *o*1, *o*2, and *o*3

*i*4-*i*3 and *i*3-*i*2: the difference of latency measured between locations of *i*4 and *i*3, and *i*3 and *i*2 in the inching test

Unit of CSA = mm^2^

*p < 0.05 in comparing the two most frequent positive sites and the negative one in inching test using analysis of variance (ANOVA) followed by Scheffe's multiple comparison procedure

### Comparison of CSAs of A-hands with those of CTS-hands by CTS symptom duration

The comparative results showed that CSAs of the "CTS-hands" with symptom duration >1 month and ≤12 months, and >12 months were significantly larger than the CSAs of "A-hands". The difference between the CSAs of the "A-hands" and the "CTS-hands" with symptom duration > 1 month was not significant. The "CTS-hands" with >12 months duration had significantly larger CSA than the "CTS-hands" with <1 month duration (Table 6).

**Table 6:** Comparison of CSAs at the 8-point of the 'A-hands' with the "CTS-hands" of different symptom durations

	A-hands		CTS-hands	
		
	(n = 50)	1 month(n = 12)	> 1 month and 12 months (n = 82)	>12 months (n = 118)
***i*4**	11.8 ± 2.4 (8-19)	14.1 ± 4.7 (8-24)	16.6 ± 5.1 (7-34)*	18.5 ± 6.9 (8-41)*
***i*3**	11.5 ± 2.3 (8-19)	12.7 ± 2.9 (8-17)	16.0 ± 5.0 (8-28) *	17.3 ± 6.0 (8-42)*^,#^
**i2**	10.9 ± 1.9 (8-16)	11.2 ± 2.6 (7-17)	12.8 ± 2.8 (7-20) *	13.1 ± 3.7 (6-29)*
***i*1**	10.7 ± 1.7 (8-15)	12.8 ± 2.0 (10-16)	13.6 ± 4.1 (7-24) *	15.0 ± 5.1 (7-34.4)*
***w***	10.7 ± 2.0 (7-17)	11.7 ± 3.3 (7-18)	14.8 ± 5.2 (7-40.4) *	16.0 ± 5.2 (7-33)*^#^
***o*1**	10.1 ± 2.2 ( 6-16)	11.2 ± 2.9 (7-16)	12.8 ± 3.2 (7-23) *	13.7 ± 3.4 (8-27.4)*
***o*2**	10.2 ± 2.1 (7-17)	10.8 ± 2.0 (8-15)	11.6 ± 2.3 (6-19) *	12.7 ± 2.8 (7-21.3)*
***o*3**	9.4 ± 1.9 (6-15)	10.5 ± 2.6 (7-17)	11.1 ± 2.2 (5-16) *	11.4 ± 2.6 (6-19)*

Abbreviations: A-hand, asymptomatic hands; CTS-hands, carpal tunnel syndrome hand; CSAs, cross section areas Markers of the 8-point: *i*4, *i*3, *i*2, i1, *w*, *o*1, *o*2, and *o*3. Unit of CSA = mm^2^

*p < 0.05 (a comparison of CSAs of the 'A-hands' with the CSAs of the 'CTS-hands' of different symptom durations using analysis of variance (ANOVA) followed by Scheffe's multiple comparison procedure)

^#^*p *< 0.05 (a comparison of CSAs of the CTS-hands with <1 month duration with the CSAs of the 'CTS-hands' with >12 months duration using analysis of variance (ANOVA) followed by Scheffe's multiple comparison procedure)

## Discussion

For CTS evaluation, several kinds of NCS measurement methods are used for confirmation. As to which measurement method is optimum remains the subject of, long-term debates [1-6,10-20]. In the meantime, although NCS in CTS diagnosis is highly specific [2], 10-25% of cases are unrecognized by classic NCS depending on the disease severity and the type of NCS technique used [2,21-23]. Thus, "CTS-hands" with a negative NCS poses a diagnostic challenge when using electrophysiologic study alone for confirmation.

In this study, 15.3% (32/212) of "CTS-hands" are NCS negative. This incidence rate is consistent with those of previous reports [2,21-23]. With the 8-point CSA measurement, there are significant differences on several levels between the "A-hands" and NCS negative "CTS-hands". Most of the significant enlargements are located at the inlet (Table 2). It is known that in patients with a clinical diagnosis of CTS, the accuracy of US is similar to that of EMG but is probably preferable because it is painless, easily accessible, and favored by patients [24]. The findings of the present study further strengthen the importance of the complementary role of US in confirming the diagnosis of idiopathic CTS in the NCS negative group. This is also noted in a study of US correlation of CTS in NCS negative "CTS-hands" reported by Rahmani et al. [19]. Therefore, US can be recommended as a useful technique in diagnosing CTS patients when NCS results are not confirmatory in patients suspected of having median neuropathy. The present study also posits the following cut-off values of CSA for CTS confirmation: 12.5 mm^2 ^at the tunnel inlet, 11.5 mm^2 ^at the wrist crease, and 10.1 mm^2 ^at the tunnel outlet.

Except for the NCS minimal group, all of the other groups of "CTS-hands" (from mild to extreme) have significant differences in CSA measurement when compared to that of the NCS negative group (Table 3 and Figures 2 and 3) and a positive correlation with the severities of NCS findings. Although some insignificant enlargements detected in CSA measurement are shown by inter-group comparison (Tables 2 and 3), the present study demonstrates that slower NCS means a larger CSA by US study.

As shown in Table 3 and Figures 2 and 3, CSAs measured at the 8-point of the NCS minimal group are all larger than those of the NCS negative group, but this difference is not statistically significant. This insignificance can be explained partly by the trivial difference in NCS and measured CSAs in these two groups of "CTS-hands". However, this study does not offer enough evidence to sufficiently explain the difference. Further large-scale study is needed for better delineation of the US findings between the NCS negative and NCS minimal groups. Nevertheless, with a measurement of CSA at the 8-point, US remains an important complementary tool for confirming clinical CTS.

As shown in Table 4, the segments between *i*4 and *i*3, and *i*3 and *i*2 are the most frequent "positive sites", and their respective CSAs are larger than those measured at "non-positive sites" (Table 5). This suggests a positive correlation in NCS severities and measured areas of CSA in the CTS study, a correlation also noted in other studies [10,20,25,26]. The present study (Tables 4 and 5) also reveals that most of the "positive sites" detected in the "inching test" involve the distal part (*i*2-*1*4) of the inlet, and the CSA measured at *i2 *is the smallest. These show that the area around *i*2 is the most possible site of nerve entrapment in idiopathic CTS, which may provide additional guidance for a more precise location for treatment.

As shown in Table 6, there is a positive correlation between the measured CSA with the symptom duration of clinical idiopathic CTS such that the longer the duration of symptoms correlated to larger measured CSA. This finding has not been previously reported. Nonetheless, US provides reproducible median nerve measurements [27]. As such, it can be used to assess changes in median nerve characteristics during follow-up studies of idiopathic CTS.

This study has several limitations. First, although 212 "CTS-hands" were included for examination, further large-scale study is warranted for a more even distribution of the case number in the different sub-groups of "CTS-hands". Second, the limitations of accuracy in inching techniques need to be taken into consideration. This limitation is also noted in other studies [28,29]. Third, there is difficulty in accurately obtaining a chronology of the length of symptom duration. Fourth, there is a lack of using neuroimaging studies such as computed tomography and/or magnetic resonance imaging to test the accuracy of CSA measurement at varying levels and to delineate the local change of carpal tunnel. Fifth, besides CSA measurement, there are other useful, additional measurements of the median nerve with US such as the measurement of width and circumference of the wrist [30]. In this study we did not perform these additional measurements for CSA correlation. Lastly, there is a discrepancy of median nerve length between the conventional surface measurement and US measurement [31].

## Conclusions

More than 15% of "CTS-hands" have negative NCS. The 8-point measurement of the median nerve CSA from inlet to outlet similar to the "inching test" provides more information on anatomic changes. This US finding has positive correlation with NCS severity and the duration of CTS clinical symptom. A combination of NCS and US studies, especially the 8-point measurement, may have a higher positive rate than NCS alone for diagnosing CTS

## Competing interests

The authors declare that they have no competing interests.

## Authors' contributions

All authors have read and approved the submitted manuscript.

SFC contributed to the conception and design, data acquisition and analysis, and drafting and revision of the manuscript; CHL, CRH, YCC, NWT, and CCC contributed to the conception and design, and clinical data analysis; and WNC contributed to the conception and design, data analysis, and critical revision and final approval of the manuscript.

## Pre-publication history

The pre-publication history for this paper can be accessed here:

http://www.biomedcentral.com/1471-2342/11/22/prepub
